# The Incidence of Perioperative Cardiac Events after Orthopedic Surgery: A Single Institutional Experience of Cases Performed over One Year

**DOI:** 10.1007/s11420-017-9561-9

**Published:** 2017-07-31

**Authors:** Michael K. Urban, Steffan W. Wolfe, Neil M. Sanghavi, Kara Fields, Steven K. Magid

**Affiliations:** Hospital for Special Surgery, 535 East 70th Street, New York, NY 10021 USA

**Keywords:** postoperative myocardial ischemia, orthopedic surgery, postoperative cardiac complications, ischemic heart disease

## Abstract

**Background:**

Orthopedic patients with ischemic heart disease are at risk for postoperative cardiac complications.

**Questions/Purposes:**

Using information from two medical information retrieval systems which insured the capture of all events for the period of study, our goals were to determine the incidence of myocardial injury in at-risk patients after orthopedic surgery and to delineate the type and incidence of cardiac complications in this population.

**Methods:**

For one year, at an orthopedic hospital, we identified all postoperative patients with a measured cTnI level using an electronic ordering system. Preoperative cardiac risk factors and postoperative cardiac complications were identified in patients undergoing a total hip arthroplasty (THA), total knee arthroplasty (TKA), and posterior spinal fusion (PSF). A postoperative myocardial infarction was defined by a cTnI > 0.1 ng/mL, ECG changes, new echocardiographic regional wall motion abnormalities, and evaluation by a cardiologist. Categorical variables were compared among groups with a Fisher’s exact or Chi-square test. Continuous variables were compared among groups with ANOVA or the Kruskal-Wallis test. The associations of cardiac risk factors with myocardial injury are expressed as odds ratios from logistic regression models.

**Results:**

During a one-year period, from 10,627 inpatient orthopedic procedures, 805 patients were identified as at risk for postoperative myocardial ischemia. A total of 20.6% (166/805) of these patients had elevated serum cTnI levels (cTnI > 0.02 ng/mL), and there were ten documented postoperative MIs (10/805; 1.2%). For the at-risk TKA, THA, or PSF patients, 19% (102/532) had elevated cTnI levels and 31% (32/102) had postoperative cardiac complications, including arrhythmias (56%), congestive heart failure (2%), and MI (1%). Adjusting for sex, age, BMI, cardiac risk factors, and medications (statins and β-blockers), PSF patients had 3.9 times the risk of myocardial injury (*p* = 0.003) compared to TKA patients and 4.2 times that of THA patients.

**Conclusions:**

The incidence of postoperative myocardial ischemia after major orthopedic surgery in patients with cardiac risk factors is high (8.7%), but the incidence of documented myocardial infarctions and serious cardiac complications remains low (1.2–2%). Patients with higher postoperative cTnI releases were more likely to have cardiac complications, and some procedures (spinal fusions) placed the patients at a higher risk.

**Electronic supplementary material:**

The online version of this article (doi:10.1007/s11420-017-9561-9) contains supplementary material, which is available to authorized users.

## Introduction

As our population ages, there will be a demand for a continued active lifestyle. Concomitantly, there will be an increase in demand for orthopedic surgery. In the United States, one in five adults has been diagnosed with osteoarthritis CDC: Morbidity and Mortality Weekly Report 2016 (MMWR). By 2030, there may be 500,000 total hip arthroplasty (THA) and 3 million total knee arthroplasty (TKA) cases per year [5]. Orthopedic surgery is associated with a number of conditions which impose significant physiologic stresses [6]. These include significant blood loss with concomitant fluid shifts, promotion of an inflammatory response due to the embolization of fat and other substances from the bone, and significant postoperative pain with its associated adrenergic correlates. In addition, joint arthroplasty procedures are increasingly performed in older patients with increased medical morbidities. Patients with ischemic heart disease (IHD) have a significant risk of cardiovascular complications and mortality after major non-cardiac surgery [7, 14, 22, 24]. This may explain the higher incidence of cardiac complications reported after some types of orthopedic procedures [2, 16, 21, 23].

The mortality associated with myocardial infarction after hip and knee arthroplasty surgery ranges from 0.4 to 4.6% [12, 17, 21]. Furthermore, cardiac complications are most often associated with perioperative mortality. In their review of 1636 consecutive hip and knee replacements, Parvizi et al. reported a 6.4% incidence of serious postoperative complications, the majority of which were cardiac [20]. Therefore, given the aging of the population, the increasing rates and feasibility of surgery in the elderly and the current prevalence of perioperative cardiovascular morbidity and mortality, it should be anticipated that postoperative cardiac complications will remain an important public health concern. A publication from the Netherlands reported a 25-fold increase in postoperative myocardial ischemia (PMI) within two weeks after THA and 31-fold increase after TKA compared with that of the matched non-surgical patients [13].

Early detection of postoperative cardiac injury allows proactive intervention which may in turn improve outcome because these events are often associated with cardiac morbidity if not treated appropriately. Furthermore, the decision to initiate postoperative physical therapy, which is important for a favorable outcome in orthopedic patients, may depend on the correct diagnosis of postoperative myocardial ischemia (PMI).

The introduction of plasma troponin I (cTnI) analysis has markedly increased our ability to detect postoperative myocardial events because plasma elevations in this protein are highly suggestive for cardiac injury. In addition, cTnI has been shown to be a more specific marker for PMI than creatine phosphokinase (CPK) after orthopedic surgery [1, 11, 25].

In this report, we assessed the incidence of cTnI elevations in patients who have risk factors associated with IHD. There have been previous reports assessing the incidence of myocardial ischemia after non-cardiac surgery [7–10, 16–19]. We obtained this information from two medical information retrieval systems which ensured the capture of all events for the period of study; e.g., every elevated troponin during this period was matched to a patient. Our goals were to determine the incidence of myocardial injury in patients at risk after orthopedic surgery at one institution where similar procedures have similar perioperative experiences. In addition, to delineate the type and incidence of cardiac complications in this population and identify which patients and procedures are at risk for myocardial ischemia after orthopedic surgery.

## Patients and Methods

With the IRB approval, for one year (7/01/07–6/30/08), at an orthopedic hospital, we identified all postoperative patients with a measured cTnI level using an electronic ordering system; Sunrise Medical Management® (Allscripts). During this period, postoperative patients with known IHD or risk factors for IHD [15], assessed by their preoperative medical and/or cardiology consultant, were entered into a ROMI (rule-out-myocardial ischemia) protocol which included cTnI analysis on entry to the PACU (postanesthesia care unit) and 12 h later. Patients with anginal symptoms of myocardial ischemia (including chest pain, shortness of breath associated with CHF) during their postoperative period were also entered into the ROMI protocol.

An elevated troponin was defined as the value (>0.02 ng/mL) above the upper limit of the manufacturer’s reference range (Architect Stat Troponin I, Abbott Laboratories, Abbott Park, Ill). A value of ≥0.1 ng/mL is more than the 99th percentile for the normal population and is indicative of myocardial injury [26]. Patients with elevated cTnI levels continued to have levels assessed every 8 h until they decreased while they were monitored in an ICU where heart rate and blood pressure instability was treated aggressively [26].

Preoperative cardiac risk factors and postoperative cardiac complications were identified using a web-based medical information management system, My Medical Files, MMF^®^. These risk factors and complications were identified in patients undergoing a total hip arthroplasty (THA), total knee arthroplasty (TKA), and posterior spinal fusion (PSF), the most common inpatient orthopedic procedures performed at this institution. The preoperative risk factors which were evaluated included the following: a prior myocardial infarction, inducible myocardial ischemia, treated angina, the presence of coronary artery stents, diabetes mellitus, renal insufficiency (Cr > 2.0), a history of congestive heart failure (CHF), and a history of a cerebral vascular accident (CVA) [15]. Patients treated with β-blockers and statins were also identified. All cardiac risk patients with elevated troponins were treated with 81 mg aspirin daily. ICD-9 diagnostic codes for dysrhythmias, congestive heart failure, and myocardial infarction were included as postoperative cardiac complications. Patients with elevated troponins had continuous cardiac monitoring for the first postoperative 24 h. However, once stable and without rising troponins, the detection of cardiac arrhythmias was dependent on the detection by routine medical care. Postoperative myocardial infarction was confirmed by ECG changes and the presence of echocardiographic (trans-thoracic echocardiogram, TTE) regional wall motion abnormalities (RWMAs). When possible (patients with a previous MI and/or positive history of myocardial ischemia), the RWMAs on the postoperative TTE were compared to those of the preoperative TTE. For those patients with PMI and postoperative cardiac complications, the patient’s medical chart was reviewed to insure the accuracy in reporting. All patients with evidence of a myocardial infarction were evaluated by a cardiologist and when appropriate transferred to a cardiac care unit (CCU) for evaluation and possible emergency cardiac catheterization and angioplasty. For this analysis, a postoperative myocardial infarction was defined as including all of the following: elevated troponins, new ECG changes, RWMAs on an echocardiogram, and agreement by the consulting cardiologist.

Categorical variables are reported as frequencies and percentages and were compared among groups with a Fisher’s exact or Chi-square test. Continuous variables were summarized as means and standard deviations or medians and interquartile ranges and compared among groups with ANOVA or the Kruskal-Wallis test. The associations of cardiac risk factors, procedure type, and medications with postoperative myocardial injury are expressed as odds ratios and 95% intervals derived from logistic regression models. All statistical tests were two-sided and *p* values less than 0.05 were considered statistically significant. The data were analyzed with the SAS version 9.3.

No external funding, apart from the support of the authors’ institution, was provided for this study. The study was performed under the auspices of the HSS Quality Research Center.

## Results

**Fig. 1:**
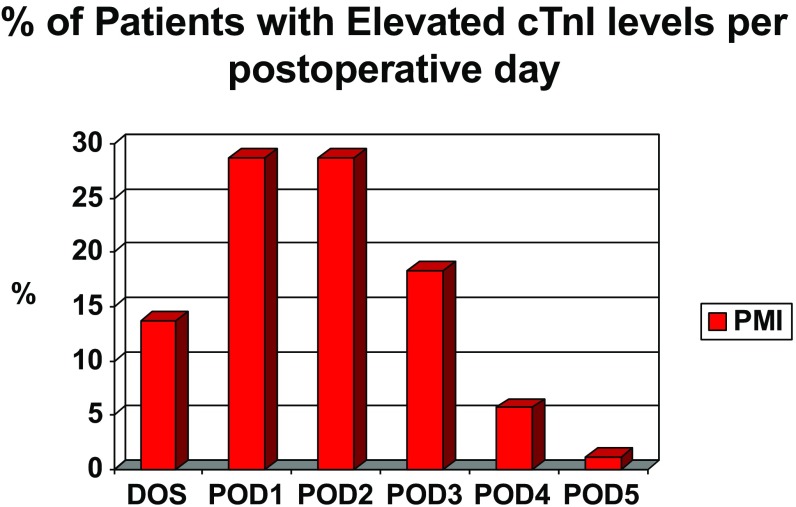
% of patients with elevated cTnI levels per postoperative day.

During a one-year period, 10,627 inpatient orthopedic procedures were performed at a single musculoskeletal hospital. For all major orthopedic procedures, 805 patients were identified as at risk for postoperative myocardial ischemia either by risk factors (65%) or by postoperative anginal symptoms (35%) and were then entered into a postoperative “Rule Out MI” protocol which included serial cTnI levels and electrocardiograms. Of these patients, 20.6% (166/805) had elevated serum cTnI levels (cTnI > 0.02 ng/mL) and 8.7% (70/805) had troponin releases suggestive of myocardial injury (cTnI ≥ 0.1 ng/mL). The incidence of myocardial ischemia in the asymptomatic at-risk population was 6.8% (36/530) compared to 13.1% (36/275) in the symptomatic population. The majority of the myocardial ischemic events (~90%) occurred by postoperative day 3 (Fig. 1). For the entire population of at cardiac-risk patients undergoing major orthopedic surgery, there were ten documented postoperative MIs (10/805) with an incidence of 1.2%.

Of the 805 patients identified as at risk for postoperative myocardial ischemia, 532 included patients who underwent a TKA, THA, or PSF. Demographics and cardiac risk factors for TKA, THA, and, PSF patients are shown in Table 1. Patients undergoing PSF were younger and less likely to be on β-blockers or statins compared to TKA and THA patients. Otherwise, the patients had similar co-morbidity profiles.

**Table 1:** Cardiac risk factors for TKA, THA, and PSF patients^a^.

Procedure	TKA (*n* = 209)	THA (*n* = 231)	PSF (*n* = 92)	All (*n* = 532)	*p* value
Age (SD)	74 ± 9	72 ± 8	67 ± 11	72 ± 10	<0.001
BMI (SD)	26 ± 6	31 ± 6	28 ± 5	29 ± 6	0.415
Male (%)	119 (57)	118 (51)	51 (55)	288 (54)	0.451
β-blocker (%)	119 (57)	148 (64)	4 (4)	271 (51)	<0.001
Statins (%)	85 (41)	110 (48)	6 (7)	201 (38)	<0.001
Previous MI (%)	27 (13)	24 (10)	10 (11)	61 (12)	0.699
+ Stress test (%)	12 (6)	20 (9)	2 (2)	34 (6)	0.091
Angina (%)	28 (13)	25 (11)	8 (9)	61 (12)	0.472
Stents (%)	36 (17)	35 (15)	11 (12)	82 (15)	0.520
Previous CVA (%)	5 (2)	3 (1)	1 (1)	9 (2)	0.667
DM (%)	15 (7)	23 (10)	7 (8)	45 (9)	0.554
Cr ≥ 1.6	11 (5)	10 (4)	2 (2)	23 (4)	0.487
Previous CHF (%)	3 (1)	6 (3)	1 (1)	10 (2)	0.692

^a^Data are listed as number of patients ± SD or number (%).

*THA* total hip arthroplasty, *TKA* total knee arthroplasty, *PSF* posterior spine fusion, *MI* myocardial infarction, *stents* coronary artery stents, *CVA* cerebral vascular accident, *DM* diabetes mellitus, *Cr* blood creatinine level, measured in mg/dL.

Table 2 shows the incidence of elevated cTnI, myocardial injury, MI, and CHF by procedure. For the at-risk TKA, THA, or PSF patients, 19% (102/532) had elevated cTnI levels and 8% (44/532) demonstrated evidence of myocardial injury. A higher percentage of PSF patients exhibited myocardial injury (14%) than either THA (7%) or TKA (8%) patients. Of those patients with elevated cTnI levels, 31% (32/102) had postoperative cardiac complications. The majority (56%) of which were arrhythmias, which in some cases precipitated the ischemia. However, serious complications were rare with a 1% (5/532) incidence of documented MI, as assessed by echocardiographic regional wall abnormalities, and 2% (9/532) incidence of symptomatic CHF.

**Table 2:** Postoperative complications by procedure^a^.

	TKA (209)	THA (231)	PSF (92)	All (532)
cTnI > 0.02 (%)	38 (18)	41 (18)	23 (25)	102 (19)
cTnI ≥ 0.1 (%)^b^	16 (8)	15 (7)	13 (14)	44 (8)
MI (%)	1 (1)	2 (1)	2 (2)	5 (1)
CHF (%)	2 (1)	5 (2)	2 (2)	9 (2)

^a^Data are listed as number of patients and number (%), unless stated otherwise.

^b^Myocardial injury defined as cTnI ≥ 0.1 ng/mL.

*THA* total hip arthroplasty, *TKA* total knee arthroplasty, *PSF* posterior spine fusion, *MI* myocardial infarction, *CHF* congestive heart failure

**Fig. 2:**
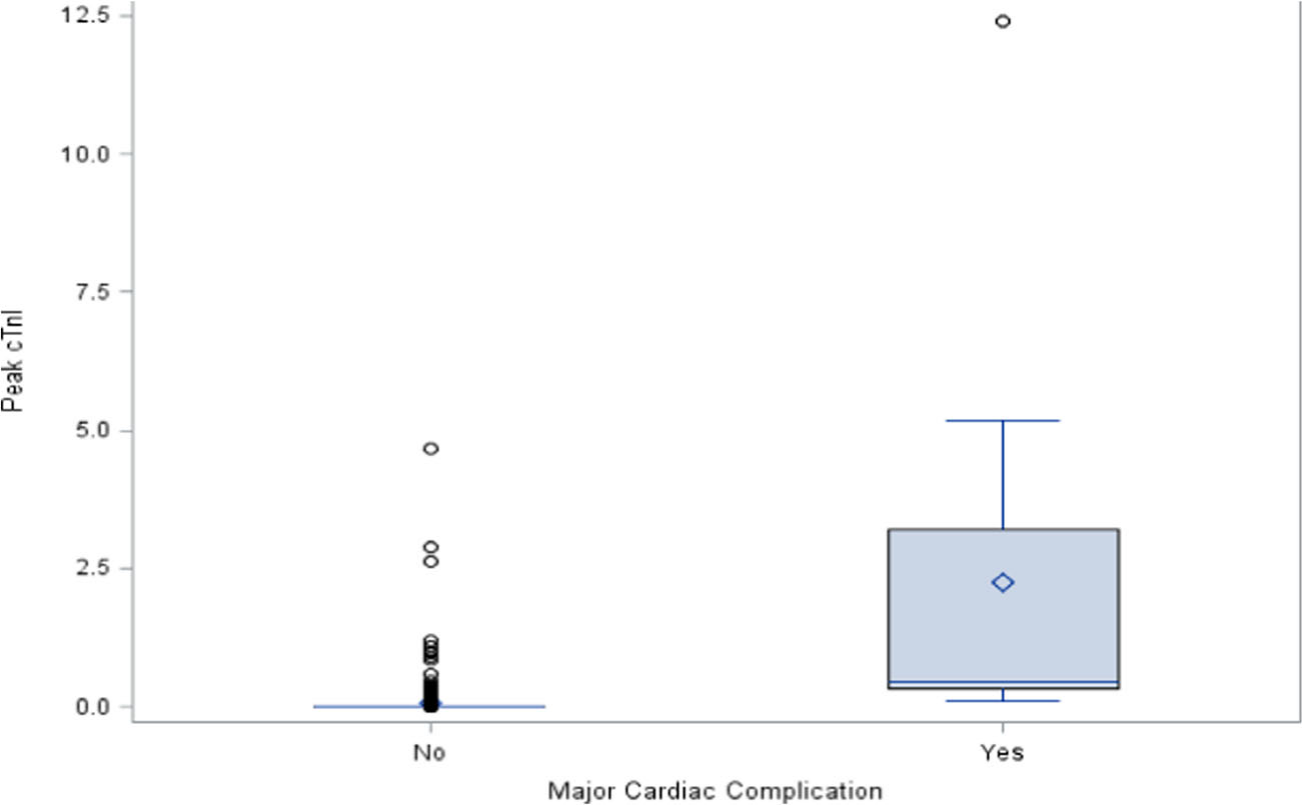
Peak cTnI levels (ng/mL) in patients with major cardiac complications. Median (IQR) peak cTn1 levels for patients who did not experience major cardiac complications were 0.02 ng/mL (0), whereas levels for patients who did experience complications were 0.45 ng/mL (2.89); *p* < 0.001.

Of these 102 patients with elevated cTnI levels, 41 were part of the routine postoperative ROMI protocol and 61 were assessed for myocardial ischemia due to postoperative symptoms. The median peak cTnI levels for these two groups were statistically similar (0.06 and 0.08 ng/mL). Median (IQR) peak cTnI levels were also similar for all at-risk THA, TKA, and PSF patients, while the median peak cTnI levels were 0.02 and 0.45 ng/mL (2.89) for the same patients who did and did not experience major cardiac complications, respectively (*p* < 0.001; Fig. 2).

Table 3 shows the odds ratio estimates derived from logistic regression modeling for myocardial injury. Adjusting for sex, age, BMI, multiple cardiac risk factors, and medications (statins and β-blockers), PSF patients had 3.9 (95% CI 1.5–10.4) times the odds of postoperative myocardial injury compared to TKA and 4.2 (95% CI 1.5–11.4) times the odds compared to THA patients (*p* = 0.003). There was no evidence of a significant association between β-blocker administration, statin administration, or multiple cardiac risk factors and the odds of postoperative myocardial injury.

**Table 3:** Logistic regression modeling for myocardial injury.

Variable	Odds ratio (95% CI)	*p* value
Age	1.04 (1.00–1.09)	0.032
Sex (M vs. F)	1.56 (0.81–3.03)	0.185
BMI	0.99 (0.93–1.05)	0.750
Procedure: PSF vs. THA	4.17 (1.52–11.43)	0.003
TKA vs. THA	1.06 (0.49–2.29)	0.074
β-blockers	1.85 (0.80–4.32)	0.153
Statins	0.90 (0.41–1.99)	0.791
Previous MI	0.83 (0.28–2.45)	0.730
+ Stress test	0.39 (0.05–3.06)	0.373
Angina	0.57 (0.18–1.83)	0.344
Stents	2.23 (0.89–5.58)	0.087
Previous CVA	1.64 (0.18–14.77)	0.662
DM	0.42 (0.09–1.99)	0.274
Cr ≥ 1.6	1.83 (0.47–7.15)	0.387
Previous CHF	1.34 (0.15–12.18)	0.793

*PSF* posterior spine fusion, *THA* total hip arthroplasty, *TKA* total knee arthroplasty, *MI* myocardial infarction, *stents* coronary artery stents, *CVA* cerebral vascular accident, *DM* diabetes mellitus, *Cr* blood creatinine level, *CHF* congestive heart failure

## Discussion

By querying our electronic ordering system, we were able to capture all patients for one year undergoing major inpatient orthopedic procedures who were evaluated for postoperative myocardial ischemia, assessed by elevated troponin levels. Assuming that all patients with postoperative myocardial events were captured, then 1.6% (166/10627) of all patients undergoing major orthopedic surgery had postoperative elevated troponin levels and 0.7% (70/10627) demonstrated evidence of postoperative myocardial injury, PMI.

We acknowledge that this study has limitations. We assumed that all patients with significant cardiac risk factors or perioperative cardiac events were assessed for a PMI through cTnI analysis. However, it is possible that stable asymptomatic patients with postoperative myocardial ischemia went undetected during their hospitalization. All of our patients for major inpatient orthopedic procedures must undergo medical clearance, and patients with major cardiac risk factors have been routinely entered into a ROMI protocol with cTnI analysis. Furthermore, since risk factor assessment was only performed for those patients in the ROMI patient pool, we cannot assess the association for any given co-morbidity and the risk of a PMI for the entire population of surgical patients. We concentrated our analysis on the 532 patients in the ROMI pool who had undergone a TKA, THA, or PSF, but an additional 273 patients had IHD, of which 64 had elevated cTnI levels but were not entered into our detailed analysis. Furthermore, the data was obtained from a patient population undergoing surgery eight years ago. However, much of our practice has not changed much during this period and only now is undergoing reassessment based on this type of information. Also, the information systems employed at this time permitted us to analyze the necessary components for the study.

Several previous studies have reported an incidence of between 0.3 and 0.9% for orthopedic surgery [17, 20, 26]. Gandhi et al. [10] reported the PMI incidence to be 1.8% within 18 days of a THA or TKA. However, Lalmohamed et al. [13] reported the incidence to be about 0.8% in the two-week period after a TKA or THA. This is similar to our incidence of 0.7% for a PMI while in the hospital (<5 days) after a THA or TKA. Oberweis et al. [19] reported that for all patients undergoing hip, knee, or spine surgery, the incidence of “myocardial necrosis” (elevated troponins) was 5.9%, and 17% of those patients entered into a ROMI protocol. This is similar to our finding of 19% for at-risk TKA, THA, or spine fusion with elevated postoperative troponins.

As reported by others, we found that the majority of the myocardial ischemic events occurred within the first few days after surgery [10, 20]. Myocardial oxygen supply and demand imbalance is the predominant cause of myocardial ischemia during this early postoperative period [3, 8]. In the presence of coronary stenosis, the ischemia is triggered by increased myocardial oxygen demand (tachycardia, hypertension) or decreased oxygen supply (thrombosis, anemia). This may explain the higher incidence of a PMI after spinal fusion compared with other orthopedic procedures. Spinal fusion surgery is associated with increased blood loss, fluid shifts, and pain compared to arthroplasty surgery. In addition, spinal fusion patients received a general anesthetic while the arthroplasty procedures were usually performed under regional anesthesia. Regional anesthesia may provide a protective effect over general anesthesia with regard to postoperative complications [18]. One would also expect that stress reduction possibly in the form of β-adrenergic blockade would reduce the incidence of PMI. Although we were unable to demonstrate an advantage for those patients on chronic perioperative β-blockers with respect to reduced postoperative myocardial ischemia, β-blockers and statins were stopped before PSFs, and these patients had a higher incidence of PMI. Some studies support the policy of maintaining β-blockers throughout the perioperative period in order to reduce the incidence of PMI [27]. Also, anti-platelet and anti-coagulant medications are often held for several days after PSFs, while they are initiated in some from the day of surgery for THA and TKA patients.

Cardiac complications are most often associated with perioperative mortality. In their review of 1636 consecutive hip and knee replacements, Parvizi et al. reported a 6.4% incidence of serious postoperative complications, the majority of which were cardiac [20]. In the POISE trial of prophylactic perioperative β-blocker therapy for patients undergoing non-cardiac surgery (*n* = 8351), the incidence of a nonfatal MI was 4.4% and cardiovascular death 1.6% [19]. All of these patients had cardiovascular risk factors for a PMI as did the orthopedic patients assessed for a PMI in this study. However, we found that 8.7% of the patients which we identified as at risk for postoperative ischemia (IHD) had a PMI. Is an 8.7% PMI incidence among all orthopedic surgical patients with cardiac risks higher than expected? Part of the answer will rest with the interpretation of the significance of various cTnI levels. The majority of our patients had low cTnI level releases, and they were not related to major cardiac complications. Only ten of the patients had documented myocardial infarctions on the basis of an ECG and new echocardiographic changes, and only seventeen of the 805 ROMI patients with an elevated cTnI level were transferred to a cardiac critical care unit for congestive heart failure or hemodynamic instability. Using the above criteria to define a PMI, this would reduce the incidence to 1.2 (10/805) or 2.1% of all (17/805) orthopedic surgical patients with cardiac risk factors. These low event rates may possibly be related to our aggressive treatment of pain, judicious use of fluids, and interventions, particularly β-blockade, to maintain hemodynamic stability [1].

Since 8% of our surgical population with cardiac risk factors were enrolled in a rule-out-PMI protocol (ROMI) while <2% demonstrated detectable cTnI leaks, the question remains: is this an optimal use of health care resources? This finding would support a more stringent approach that would require cTnI levels only on patients with perioperative cardiac events, inducible myocardial ischemia, and coronary artery stents. Several lines of evidence suggest, however, that this may not be the best approach to identify patients with PMI [4, 6]. In addition, for those patients with cardiac risk factors, we were unable to identify which patients were more likely to have postoperative cardiac events.

In a previous publication, we demonstrated that even a low level of postoperative cTnI leaks was associated with cardiac complications within 6 months of discharge from the hospital [26]. The identification of patients with myocardial injury (cTnI > 0.1 ng/mL) was valuable, since these patients had a higher incidence of postoperative cardiac complications and warranted cardiac consultation. Furthermore, after emergency orthopedic surgery, even asymptomatic troponin elevations were associated with increased cardiac events and one-year mortality [4]. Recently, two prospective studies of patients undergoing non-cardiac surgery reported that postoperative cardiac troponin elevations were strongly associated with mortality within 30 days and 12 months of surgery [6, 8]. This would suggest that patients with significant cardiac risk factors should be assessed for perioperative ischemia in order to formulate a plan for the best perioperative medical care.

In conclusion, although the incidence of postoperative myocardial ischemia (defined by an elevated cTnI) after major orthopedic surgery in patients with cardiac risk factors is high (8.7%), the incidence of documented myocardial infarctions and serious cardiac complications remains low (1.2–2%). Patients with higher postoperative cTnI releases were more likely to have cardiac complications, but we were unable to identify which patients with IHD were more likely to have PMI, except that some procedures (spinal fusions) placed the patients at a higher risk. Hence, we believe there is value in measuring postoperative troponin levels in patients with IHD, since it provides clinicians with information with regard to further management of these patients (such as transfer to a CCU or cardiac catheterization) with possibly improved outcome.

## Electronic supplementary material

ESM 1 (PDF 1224 kb)

ESM 2 (PDF 1224 kb)

ESM 3 (PDF 1224 kb)

ESM 4 (PDF 1224 kb)

ESM 5 (PDF 1224 kb)
